# Development and Characterization of Bioactive Glass Containing Composite Coatings with Ion Releasing Function for Antibiotic-Free Antibacterial Surgical Sutures

**DOI:** 10.3390/ma12030423

**Published:** 2019-01-30

**Authors:** Francesca E. Ciraldo, Kristin Schnepf, Wolfgang H. Goldmann, Aldo R. Boccaccini

**Affiliations:** 1 Department of Materials Science and Engineering, Institute of Biomaterials, University of Erlangen-Nuremberg, Cauerstraße 6, 91058 Erlangen, Germany; francesca.elisa.ciraldo@fau.de (F.E.C.); kristin_schnepf@yahoo.de (K.S.); 2 Institute of Biophysics, Department of Physics, University of Erlangen-Nuremberg, Henkestraße 91, 91052 Erlangen, Germany; wgoldmannh@aol.com

**Keywords:** Zinc, silver-doped mesoporous glass, chitosan, PCL, Vicryl Plus suture, dip coating

## Abstract

Resorbable (Vicryl^®^ Plus) sutures were coated with zinc-doped glass (Zn-BG) and silver-doped ordered mesoporous bioactive glass (Ag-MBG) particles by a dip coating technique. A multilayer approach was used to achieve robust coatings. The first coating was a polymeric layer (e.g., PCL or chitosan) and the second one was a composite made of BG particles in a polymer matrix. The coatings were characterized in terms of morphology by scanning electron microscopy (SEM), in vitro bioactivity, and antibacterial properties. Chitosan/Ag-MBG coatings showed the ability to form hydroxyl-carbonate-apatite on their surfaces after immersion in SBF. An antibacterial effect against Gram (+) and Gram (-) bacteria was confirmed, highlighting the potential application of the coated sutures for antibiotic-free approaches.

## 1. Introduction

Due to their widespread application in medicine, e.g., routine skin laceration or organ transplantation [1,2], sutures are one of the largest groups of medical devices implanted in humans (250 million per year only in the USA) [3]. Sutures can be produced using natural or synthetic materials and can be resorbable or non-resorbable [4]. Prominent synthetic bio-resorbable materials used for sutures are polyglycolic acid (PGA), polylactic acid (PLA), or polyglycolide lactide copolymers (PLGA). One example for PLGA sutures is Vicryl^®^ (Ethicon Inc., Edinburgh, Scotland), a widespread braided suture made of polyglactin 910, copolymer of glycolide, and lactide moieties at a ratio of 90:10. Sutures like Vicryl^®^ are often used because of their good mechanical properties, high reproducibility, and minimal tissue reaction. To further strengthen the bond between suture and tissue, several researchers have investigated the coating of sutures with bioactive substances such as bioactive glass [1,5,6,7,8]. Bioactive glasses (BGs) have been shown to bond strongly to bone as well as to soft tissues [9,10,11]. Stamboulis et al. [7] coated biodegradable sutures with bioactive glass (45S5 BG) to tailor biodegradability, to avoid a heterogeneous degradation and to improve the mechanical properties of the suture when exposed to body fluids. Pratten et al. [1] coated Mersilk^®^ (Ethicon Inc., Edinburgh, Scotland) sutures with Ag-doped BG by a conventional slurry-dipping technique. Ag-doped BG was used to make the suture bioactive and antibacterial. The coated sutures showed antibacterial properties against *Staphylococcus epidermidis*, limiting the bacteria adhesion to the surface of the samples. The use of antibacterial bioactive glasses is being considered to replace the controversially discussed triclosan [7,12,13,14] used in Vicryl^®^ Plus (Ethicon Inc., Edinburgh, Scotland) sutures.

The introduction of a foreign device in the human body can induce inflammatory reactions or introduce pathogens on site, which in turn could cause infections. Studies have shown that in 2%–5% of all surgeries, surgical site infections (SSIs) can occur [6] with consequent delays in healing, inconvenience for the patient, and in some extreme cases, even leading to death. In addition, SSIs are estimated to cause extra healthcare costs of 1.5 billion dollars just in the USA alone [1,6]. Recent studies have proposed the use of local drug delivery systems to limit bacterial infection after surgeries [15]. The introduction of a local drug delivery system offers a reduction of toxicity and side effects and the possibility to have an effective, highly controlled release. Mesoporous bioactive glasses (MBGs), first reported in 2004 [16], have been proposed as optimal candidates as local drug delivery vehicles. Thanks to their textural properties (e.g., large surface area, ordered mesoporosity with pores in a range of 2–50 nm) MBGs can be used for the release of biologically active molecules with specific effects on cells [17,18,19,20,21].

In the present work, composite sutures were produced combining commercially available sutures (Vicryl^®^ Plus) with silver containing ordered mesoporous bioactive glass (Ag-MBG) and with melt derived BG-doped with zinc (Zn-BG) particles applying, a dip coating technique. To improve the adhesion of the BG particles on the surface, two different polymers were used: chitosan and poly(ε-caprolactone) (PCL) and layered coatings were produced. Sutures were characterized in terms of acellular in vitro bioactivity by immersion in simulated body fluid (SBF) and antibacterial properties against Gram-positive *Staphylococcus carnosus* and Gram-negative *Escherichia coli*. The knot test was also performed to verify the stability and attachment of the coatings to the substrate. The BG/polymer-coated sutures developed in this study represent an innovative advance from the first BG-coated sutures reported earlier [5,6,8]. The novelty of this work is indeed the use of ordered mesoporous glass particles, which can be further loaded with growth factors or drugs (e.g., antibiotics or anti-inflammatories), and by the use of a layered polymeric matrix able to improve the adhesion of the BG particles on the surface and to modulate the release of antibacterial ions.

## 2. Results

### 2.1. Microstructural Observations

**Figure 1:**
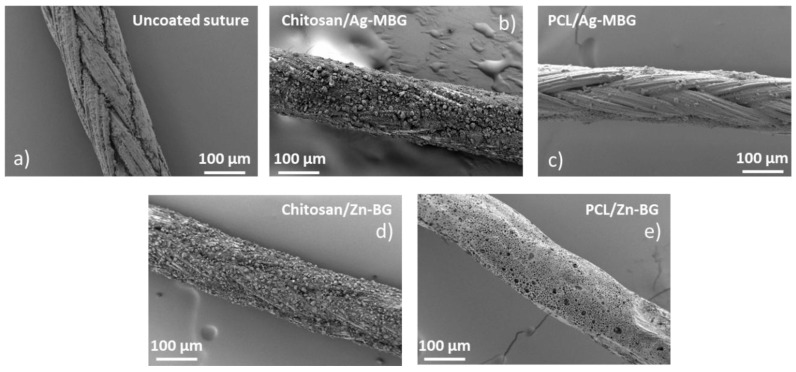
SEM micrographs showing the morphology of: (**a**) Uncoated Vicryl^®^ Plus suture, (**b**) chitosan/Ag-MBG-coated suture, (**c**) PCL/Ag-MBG-coated suture, (**d**) Chitosan/Zn-BG-coated suture, and (**e**) PCL/Zn-BG-coated suture.

The slurry dipping technique was optimized for the coating of Vicryl^®^ sutures with Ag-MBG or Zn-BG particles. Qualitative analyses of the morphology and uniformity of the coatings were performed by visual inspection and SEM observation of the glass particles attached on the surface of the samples. Figure 1 shows SEM micrographs of “as-received” and coated sutures. SEM observation confirmed that all coatings homogeneously covered the surface of the sutures.

**Figure 2:**
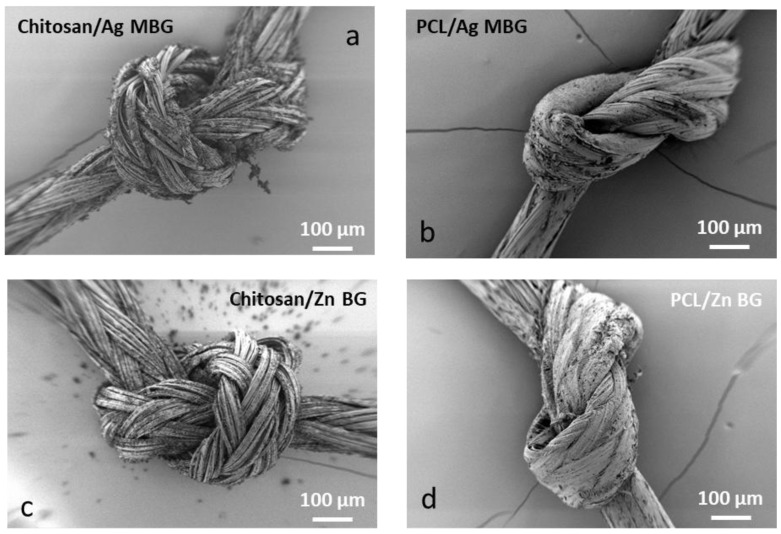
SEM micrographs of coated sutures after the knot test. (**a**) Chitosan/Ag-MBG, (**b**) PCL/Ag-MBG, (**c**) Chitosan/Zn-BG, and (**d**) PCL/Zn-BG.

The adhesion strength of the BG particles to the suture surface was not quantitatively evaluated. However, the coating stability was assessed by performing a knot test. SEM observation revealed that the majority of the coating was detached after the knot test, suggesting that the glass particles weakly adhered to the suture surface. Parts of the coating were peeled off, more at the chitosan/BG composite coating. On the other hand, the PCL/BG composite coating was more rigid and it was harder to perform the test. SEM micrographs documenting this behavior are shown in Figure 2.

### 2.2. Bioactivity Tests

**Figure 3:**
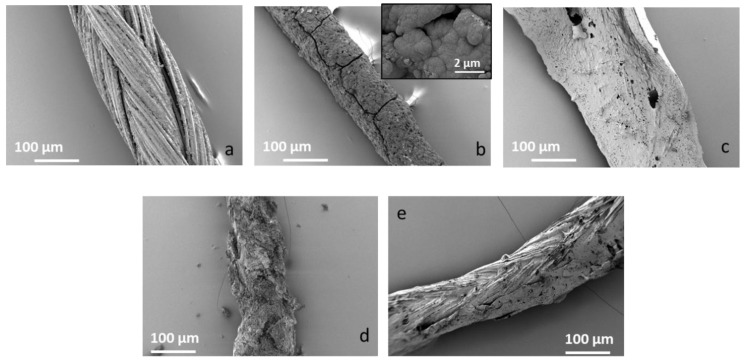
SEM micrographs of uncoated Vicryl^®^ Plus sutures. (**a**) Chitosan/Ag-MBG coated suture, (**b**) PCL/Ag-MBG-coated suture, (**c**) chitosan/Zn-BG suture, (**d**) PCL/Zn-BG suture, and (**e**) suture after 3 days of immersion in SBF: The chitosan/Ag-MBG-coated suture developed a layer, which can be ascribed to hydroxyl-carbonate-apatite based on its morphology.

In vitro bioactivity tests were performed by soaking the sutures in SBF for up to seven days. Results after three days are shown in Figure 3. After three days of immersion in SBF, the chitosan/Ag-MBG-coated suture was homogeneously covered by a layer, which appears to be hydroxyl-carbonate-apatite (HCA). On the other hand, PCL/Ag-MBG and PCL/Zn-BG-coated sutures did not show the presence of HCA even after 28 days of immersion in SBF. This result could be explained by the low amount of particles observed on the surface of the PCL samples. Moreover, similar results were shown by Miola et al. [22], who tested the ability of powdered Zn-MBG to form HCA, soaking it in SBF for up to 28 days, without finding any evidence of HCA formation.

### 2.3. Antibacterial Tests

**Figure 4:**
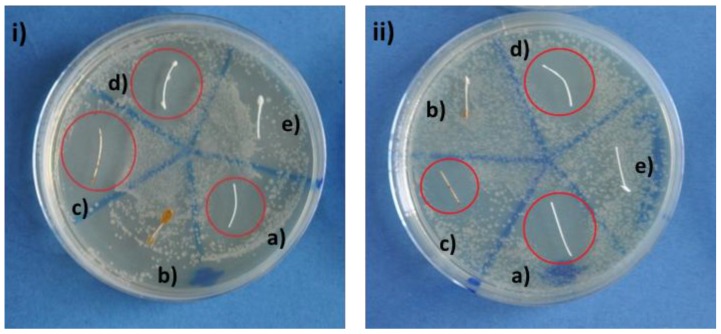
Agar-disk diffusion test with (**i**) Gram-positive (S. carnosus) and (**ii**) Gram-negative (E. coli) bacteria with (**a**) uncoated VICRYL^®^ Plus, (**b**) PCL/Ag-MBG, (**c**) chitosan/Ag-MBG, (**d**) chitosan/Zn-BG, and (**e**) PCL/Zn-BG.

Qualitative antibacterial tests with coated sutures were performed. Agar diffusion tests were carried out to assess the antibacterial properties of the coated sutures against both Gram-positive and Gram-negative bacteria (Figure 4). Uncoated sutures were taken as reference. After 24 h of incubation at 37 °C and high humidity (~80%), an inhibition halo with no bacteria is clearly visible for chitosan/Zn-BG (Figure 4i,ii, sample d)) and chitosan/Ag-MBG (Figure 4i,ii, sample c)) coated sutures for both the selected strains. However, an inhibition zone is also present around the uncoated suture (Figure 4i,ii, sample a)) due to the presence of a triclosan layer in the as-received sutures. In addition, no inhibition zone was observed on samples coated with PCL and containing Ag-MBG and Zn-BG (Figure 4i,ii, samples b and e respectively). This result suggests that the polymeric coating reduces the antibacterial effect of triclosan and that the halos of PCL/BG and chitosan/BG samples are actually induced by the progressive release of Ag^+^ and Zn^2+^ from the MBG and BG particles present in the coatings, respectively. Further studies should be performed to clarify the mechanism of antibacterial activity of Vicryl^®^ Plus sutures combined with Ag^+^ and Zn^2+^ ion release.

## 3. Discussion

The aim of this work was the development of composite coatings based on Zn-BG or Ag-MBG particles embedded in a polymer matrix of either chitosan or PCL for surgical sutures. Commercially available Vicryl^®^ (Polyglactin 910) sutures were coated with chitosan/silver-doped ordered mesoporous bioactive glass (Ag-MBG), PCL/Ag-MBG, chitosan/Zn-BG, and PCL/Zn-BG, using a slurry dipping method previously applied in other studies [1,5,7]. The dipping process was optimized and uniform and homogeneous coatings were obtained in case of chitosan/BG coated sutures. On the contrary, sutures coated with PCL/BG were characterized by the presence of a lower amount of BG particles, as a consequence of the lower BG concentration used for the preparation of the slurry. The acellular in vitro bioactive behavior of the coated sutures was investigated by immersing the samples in SBF for three days. SEM observations revealed that the chitosan/Ag-MBG coated suture was able to form HCA on the surface after 3 days of immersion in SBF. Similar results were obtained by Blaker et al. [5], who coated commercially available Mersilk^®^ and Vicryl^®^ sutures with silver-doped BG. The authors observed the formation of crystalline HA after three days in SBF. In another work, Stamboulis et al. [7] corroborated that the coating of commercially available sutures with bioactive glass particles could act as a protective “shield”, affecting the extent and rate of degradation of the sutures. However, also considering the present results, further investigations are necessary to understand the effect of the thickness and microstructure of the bioactive glass coating on the overall degradation rate and strength retention of the sutures. The adhesion and stability of the coatings were tested qualitatively, performing the knot test. Both coatings revealed limited adhesion and low mechanical stability on the substrate. However, the PCL/BG coated sutures showed better adhesion, which is probably due to the presence of fewer BG particles on the surface or due to the viscosity of the PCL solution produced in this work.

The antibacterial properties of the coated sutures were tested by means of agar diffusion tests. BG-coated sutures showed clear inhibition zones, which demonstrate their antibacterial effect. However, it should be noted that also pristine Vicryl^®^ Plus sutures showed an inhibition zone. This can be explained by the fact that this type of resorbable suture is subject to a surface treatment with triclosan, a widespread antiseptic. Nowadays, the use of triclosan is being critically discussed because some studies have indicated that this compound could promote liver tumors and provoke muscle weakness [12,13,14]. For this reason, in the last years, researchers have focused their attention on the development of new materials doped with biologically active ions (e.g., Fe, Cu, Ag, or Ga) able to limit bacteria adhesion and proliferation [23,24,25]. Nevertheless, it should be noted that the sutures coated with PCL/BG did not show any antibacterial effect against both Gram-positive and Gram-negative bacteria. It might therefore be possible that the coating isolates the triclosan layer, hindering its antibacterial effect. For this reason, it can be ascertained that the antibacterial effect observed for chitosan coatings is due to the well-known bactericidal effect of silver and zinc ions and not to the presence of triclosan. The antibacterial activity of β-chitin/ZnO nanostructured composites was already investigated by Wysokowski et al. [26]. The authors corroborated that the antibacterial activity of chitin/ZnO composite could be caused by the formation of reactive oxygen species in the presence of light. The formation of these active radicals (e.g., OH^−^ or H_2_O_2_) can provoke the oxidative stress in the bacteria, leading to perturbation of the cell membrane and damage of cell proteins and DNA [26]. The antibacterial activity of silver ions has been extensively investigated. Studies [8,24,27] have shown that Ag^+^ can cause the detachment of the cytoplasm membrane of bacteria from the cell wall, compromising the bacteria’s ability to replicate [27,28]. Moreover, bioactive glasses doped with Zn and their possible application as scaffolds for bone tissue engineering, bone filling granules, bone cements, and coatings for orthopedic applications have been widely investigated [29]. Studies [30,31] have also shown the great antimicrobial properties of Zn against both Gram (+) and Gram (-) bacteria.

However, further tests should be carried out to better understand the antibacterial mechanism of the present antibacterial sutures, in particular the possible combined effects of BGs and triclosan, which may lead to a reduction or even elimination of triclosan to achieve the desired antibacterial effect.

## 4. Materials and Methods

Undyed braided 4-0 Vicryl^®^ Plus sutures (Ethicon Inc., Edinburgh, Scotland) were used. The material is braided from fine filaments of Polyglactin 910, a copolymer of glycolide and lactide at a ratio of 90:10. The braided sutures are additionally coated with Irgacare^®^ MP (Triclosan). The sutures were cut into pieces of 1 cm length (the dimension was measured by means of a caliper) and they were coated by either zinc-doped melt derived BG (ZnBG, composition: 46.13 mol.% SiO_2_, 24.35 mol.% Na_2_O, 2.60 mol.% P_2_O_5_, 20.91 mol.% CaO, 6 mol.% ZnO) or silver-doped mesoporous ordered BG (Ag-MBG, composition: 78.00 mol.% SiO_2_, 1.20 mol.% P_2_O_5_, 20 mol.% CaO, 0.8 mol.% AgO), developed in previous works [22,27]. Chitosan (medium molecular weight 190-310 kDa, with 75–85% deacetylation degree) and poly(ε-caprolactone) (PCL, average M_n_ 8000) were purchased from Sigma-Aldrich (Schnelldorf, Germany) and used without further purification. A two-step coating was used to improve the adhesion between suture and coating; the first layer consists of a polymeric layer (chitosan or PCL) and the second one was a mixture of chitosan or PCL and Ag-MBG or Zn-BG particles. A slurry dipping method was performed manually in a glass beaker placed on a magnetic stirrer. An optimization process based on trial-and-error was used to determine the optimal composition of the slurry required to obtain uniform and homogeneous coatings.

The first layer was obtained by dipping the suture for 1 min in an aqueous chitosan (with 4% *v*/*v* acetic acid and 2% *w*/*v* chitosan) or PCL solution (with 50% *v*/*v* formic acid, 50% *v*/*v* acetic acid and 15% *w*/*v* PCL) and letting them dry for 24 h at room temperature. The second layer was applied by dipping the suture for 2 min into a suspension made of BG powder and chitosan/PCL. The second layer of chitosan/BG was prepared as follows: the chitosan solution mentioned beforehand was mixed with an aqueous slurry containing 40 wt.% BG (mixed for 2 h) and then stirred for 4 days. On the other hand, the second layer of PCL/BG was prepared by adding to the afore-mentioned PCL solution 30% *w*/*v* (with respect to PCL) of BG particles and the resultant suspension was stirred for 2 h. All slurries were produced by using benign solvents, which led to an increase in preparation time but enabled a safer work environment and may lead to a better biological compatibility of the coatings.

The microstructure and uniformity of the coatings were investigated using a light microscope (Leica M50 and IC80, Application Suite LAS V3.8 software, Leica Microsystems GmbH, Wetzlar, Germany) and scanning electron microscopy (SEM) (Gemini, Auriga, Carl Zeiss AG, Jena, Germany). The ability of coated and non-coated Vicryl^®^ sutures to form hydroxyl-carbonate-apatite (HCA) once in contact with biological fluids was assessed by immersion in simulated body fluid (SBF) for different time periods. The standard procedure described by Kokubo et al. [32] was used to carry out these experiments. Samples were placed on CellCrowns™ (Scaffdex Ltd., Tampere, Finland) inserts and immersed in 6 mL of SBF for up to 3 days. Once removed from incubation, the samples were rinsed with deionized water and left to dry at room temperature. The adhesion and stability of the coating were qualitatively evaluated by performing a knot test. The following operations were performed: threading through the eyes of surgical needles, tying a surgical knot, and bending the extremes of the sutures. After these operations, the surface of the samples was observed by SEM. The antibacterial properties of the coated sutures were evaluated using agar diffusion tests against *Escherichia coli* (Gram-negative) and *Staphylococcus carnosus* (Gram-positive). These bacteria were chosen because they are common bacteria responsible for infections [33] and they enable the direct comparison between Gram-positive and Gram-negative strains. The bacteria were obtained from the Microbiology Department of the University of Erlangen-Nuremberg, where they were routinely isolated and characterized. The bacteria population was suspended in LB (lysogeny broth) medium and its optical density (O.D.) was adjusted (at 600 mm, Biophotometer Plus, Eppendorf AG, Hamburg, Germany) to reach the value of 0.015. Then, 20 µl of the prepared medium was deposited and spread homogeneously onto a Petri dish of 10 cm diameter, which was previously covered with a uniform layer of LB-Agar. The samples (sutures) of 1.5 cm length) were placed on top and incubated overnight at 37 °C and at high relative humidity (~80 °C). The next day, the halo of the bacterial growth inhibition zone was evaluated optically and computed.

## 5. Conclusions

Surgical sutures were successfully coated by a two-step coating process to improve the adhesion between suture and coating: The first layer consisted of a polymeric layer (chitosan or PCL), and the second one formed by a mixture of chitosan or PCL and Ag-MBG or Zn-BG particles. Ag-MBG coated sutures showed a high reactivity once in contact with simulating body fluid, developing a layer of HCA after three days of immersion, while Zn-BG did not lead to HCA formation. Moreover, the chitosan coated samples showed promising results in terms of antibacterial properties against both Gram-positive and Gram-negative strains. Coatings with PCL did not show any antibacterial properties, which might be due to the low glass concentration present in the outer layer of the coating. Future investigations to determine the mechanical properties of coated sutures should be performed, e.g., by combining both polymers, in different layer structures and by optimizing the BG content.
